# Critical Incidents Scale for Ambulance Work – Denmark (CISAW-D): the development of a screening tool for work exposure to critical events in operative ambulance personnel

**DOI:** 10.29045/14784726.2022.12.7.3.26

**Published:** 2022-12-01

**Authors:** Jesper Pihl-Thingvad, Maria Louison Vang, Sara Rosenbeck Møller, Nina Beck Hansen

**Affiliations:** Odense University Hospital; University of Southern Denmark; Odense University Hospital; University of Southern Denmark; Odense University Hospital; University of Southern Denmark; University of Southern Denmark

**Keywords:** critical incidents, PTSD, work

## Abstract

**Introduction:**

Critical incidents in ambulance work are not easily compared to other risk occupations. Understanding types of incidents that can be considered critical in operational ambulance work is important to prevent work-related post-traumatic stress (PTS).

**Aim:**

This study aimed to develop a scale of critical incidents in ambulance work and assess its predictive validity in relation to the severity of PTS symptoms.

**Methods:**

A total of 1092 open-ended descriptions from Danish ambulance personnel were content analysed to develop a categorical scale that identifies types of events perceived as critical to operative ambulance personnel. Multiple regression was used to assess whether the scale predicted PTS symptoms and to assess the cumulative effect of exposure to these events.

**Results:**

The study found that the 1092 descriptions of critical events could be condensed into 28 categories of critical events. These ranged from life-threatening situations and deaths, to more daily events such as handling strong emotional reactions from patients’ relatives and working with terminally ill children. The frequency of events significantly predicted the severity of PTS symptoms with low to moderate effect (std beta = 0.2, t(375) = 3.7, p < .001), even when adjusting for known risk factors for post-traumatic stress disorder.

**Conclusion:**

This study showed that critical events in ambulance work included events that are not normally considered traumatic, and indicated that understanding the cumulative effect of these events is important when trying to prevent traumatic sequalae in ambulance personnel. The study highlighted the importance of increased focus on non-traumatic incidents that have an ongoing impact on paramedics’ mental health and well-being. The Critical Incidents Scale for Ambulance Work – Denmark (CISAW-D) is a promising tool for systematic screening for exposure to critical events in ambulance work.

## Introduction

Ambulance work entails exposure to deaths, accidents and illness of fellow human beings. Some operational situations constitute a critical incident, conceptualised as an operational incident that causes strong emotional reactions and affects the ability to function during or after the operational task (Mitchell, 1983).

Critical incidents are risk factors for post-traumatic stress disorder (PTSD) (Haugen et al., 2012). Therefore, mapping types of operational tasks that are experienced as potentially critical incidents in ambulance personnel is an important first step to understand how operational work might be detrimental to ambulance personnel’s mental health. Today, ambulance work is considered a high-risk occupation comparable to police officers, soldiers and firefighters (Skogstad et al., 2013). Although results from prevalence studies vary, a recent meta-analysis estimated the pooled prevalence rate of PTSD to be 11% among ambulance personnel (Petrie et al., 2018), notably higher than the estimated 4% prevalence of PTSD in the general population (Liu et al., 2017). PTSD in the context of risk professions is hard to detect, as prodromal stages of the illness are vague and unclear with alternating levels of post-traumatic stress (PTS) symptoms before full development of PTSD (Bonde et al., 2022; McFarlane, 2014). For example, PTSD with a delayed onset is more frequent in the context of high-risk occupations compared to civilians (Utzon-Frank et al., 2014), and PTSD in first responders is more often the result of prolonged periods of repeated exposure to multiple types of events (McFarlane, 2012). Also, the emotional symptoms of PTSD in ambulance personnel can have other primary expressions aside from fear, including emotions of overwhelming anger, resignation, frustration or sadness (Harvey et al., 2015). Due to these special features of occupational PTSD, it is important to address the type of operational work that can be considered as critical incidents. Identification of critical incidents may be a feasible way to detect personnel at high risk in occupations where symptom development might otherwise be overlooked. Characterisation of an operational work task that constitutes a critical event could help target organisational prevention and supportive initiatives on subgroups of personnel with a high exposure. Using a screening tool to monitor critical events would allow direction of extra support to personnel with a high exposure who are at an increased risk of developing PTS.

Systematic scales of exposure to critical incidents have already been developed within other high-risk occupations, that is police officers (Weiss et al., 2010), military (King et al., 2006) and firefighters (Monnier et al., 2002). However, no consensus has yet been reached on how to best operationalise critical events among ambulance personnel (Behnke et al., 2019; Donnelly & Bennett, 2014). Critical events in ambulance work are not necessarily equal to critical events in other first-responder groups. Both training and operational goals are specific to each first-responder group and important to understand development of overwhelming emotional reactions leading to psychological trauma. This is partly because the experience of loss of control or feeling overwhelmed due to lack of professional mastery may negatively affect professional identity, with the risk of prolonging negative reactions following trauma exposure (Brewin, 2003).

Only two studies have specifically focused on developing a measure of critical incidents in ambulance work (Behnke et al., 2019; Donnelly & Bennett, 2014). Both studies found that critical events were defined not only as situations including danger and severe deaths, but also as operational work of a more daily and low-risk quality, for example working with severely ill children or handling strong emotional reactions from patients’ relatives (Behnke et al., 2019; Donnelly & Bennett, 2014). The two studies conducted with German (Behnke et al., 2019) and US (Donnelly & Bennett, 2014) samples underscore the necessity of identifying critical events specific to the area of work.

Therefore, the aim of the present study was to develop a measure that could identify operational situations experienced as critical incidents in ambulance work, and to assess the measure’s predictive validity in relation to PTS symptoms.

## Methods

The current study was part of the larger project *You Don’t Stand Alone* studying exposure to critical events and development of PTSD among ambulance personnel.

Based on cross-sectional data, a mixed-method design was applied for the current study. First, each respondent described up to five critical events by answering an open-ended survey question. These descriptions were treated as qualitative data and content analysed to develop a scale of critical incidents relevant for ambulance personnel. The scale was then presented to a focus group for discussion to obtain face validity. Finally, quantitative analyses were carried out on the survey data to assess the association between the cumulative exposure to these critical events and PTS.

### Participants

Ambulance workers were recruited in 2019 from a large Danish ambulance service covering one of the five national regions. At the time of recruitment, there were 4000 operative ambulance personnel in Denmark, and sampling was based on an organisation covering 736 ambulance workers with operative duties. After thorough presentation of the project on the organisation’s intranet, all operative personnel (n = 736) were invited through their work email. Three consecutive reminders were sent. Upon written consent, participants were directed to an electronic survey. In total, 407 participants were recruited (55.3%) and 379 (51.5%) responded to the open-ended items on critical events with a mode of two to three examples from each respondent.

### Analysis

#### Sample characteristics

Sample bias was assessed using chi-square statistics in group comparisons between sample and the sample population in regards to gender, work function and station area of service. Missing data were assessed using Little’s missing completely at random (MCAR) test to analyse if items were missing in a systematic pattern based on all factors measured in the study. Descriptive statistics were used to assess the sample in regard to demographics, mental health and level of exposure.

#### Qualitative analysis and development of categories of critical events

Qualitative descriptions of critical events were gathered through one open-ended item: *Give up to five examples of the type of events that* you* experience as the most traumatic or emotionally taxing events in* your* career as an ambulance worker.* Critical incidents were not defined but the item was presented with an introduction of the goal of the question, namely to identify the most emotionally taxing operational tasks in ambulance work in order to enhance future preventive measures against PTS and burnout.

Based on 1092 open-ended responses, conventional content analysis was performed (Hsieh & Shannon, 2005). First, all data were read by the first and final author. Both are trained clinical psychologists in occupational trauma. During the first reading, basic familiarisation with the data was obtained. Data were then re-read, and text was highlighted when capturing key elements of perceived critical events. Next, impression notes of categories were developed, and data were re-read with the possibility of new emerging subcategories. Data categories were developed by identifying similarities between existing categories from each of the two coders. In total, four rounds of re-categorisation were performed before agreement was reached.

The final categories were presented to a group of six ambulance workers (all with a minimum of five years of seniority) to assess face validity. The group was part of the project’s practitioners’ board, but was assembled specifically to be part of face validity testing. The group was interviewed by the first author with a focus on recognisability and relevance of categories. The interview was conducted as an unstructured interview allowing for open discussion between the participants with the interviewer, ensuring a focus on recognisability and relevance of the categories, and that consensus was reached. Finally, interrater-reliability was assessed between the coding of the first and final author based on Cohen’s kappa coefficient.

#### Statistical analysis

A regression model was used to assess the final scale’s predictive qualities. Normal distribution, homoscedasticity and linearity were assessed visually with distribution graphs and residual and PP plots. Multi-collinearity was assessed with the variance inflation factor. Stepwise linear regression was used with predictors at step one – age, gender, work function, area of service, level of experience, crisis support and civil lifetime critical events – followed by step two where number of critical work events within the recent year was entered. Outcome was sum score of the measure of PTS. Data were deleted pairwise and bootstrapping with 1000 re-samplings and bias-corrected accelerated confidence intervals were used to account for the skewed distribution of PTS. All quantitative analyses were done with SPSS IBM Statistics, version 27.0 (Armonk, NY, USA).

### Measures

PTS was measured using the six symptom items from the validated international trauma questionnaire (Cloitre et al., 2018). Each symptom item was answered on a 5-point Likert scale from 0 = *not at all* to 4 = *extremely*, and overall symptom level was assessed by summarising the items. The sum scale showed good internal consistency with Cronbach’s alpha = 0.84.

Work critical events within the recent year were assessed with one item following respondents’ description of critical events. This item was formulated: *How many of these types of critical events have you encountered at work, within the recent year?* The item was answered by entering number of events.

Age was measured in whole years, and gender was measured as* male*, *female*, *non-gender* or *other gender orientation*.

Work function was included because each function rests on different levels of education and type of operational work tasks. Work function was coded based on data from the organisation and included six categories: *EMS-transporter*,* ambulance rescuer recruit*, *ambulance rescuer assistant*, *ambulance rescuer*, *paramedic* and *leader*.

Station area was included since service areas within a region can differ in regards to type and frequency of operational tasks as well as access to support from leader and colleagues. Seven station areas existed within the organisation and were coded as a categorical variable from 1 to 7 based on organisational data.

Crisis support was included because post-trauma support has repeatedly been shown to be an important predictor of PTSD (Brewin et al., 2000). The validated crisis support scale was used (Elklit et al., 2001). The scale consisted of seven items on support, scored on a Likert-style scale from 1 = *never* to 7 = *always*. Sum score was calculated and the scale showed good internal consistency, Cronbach’s alpha = 0.80.

Civilian lifetime critical events were included, since previous exposure to trauma has been shown to increase risk of post-traumatic reactions (Brewin et al., 2000). Civilian critical events were measured with the scale from the national co-morbidity study (Kessler et al., 1995). The scale covers a total of 14 potentially traumatic events. Answers were given as *yes/no* to each event, and were summed to assess the frequency of lifetime civilian critical events.

## Results

### Sample characteristics

The sample was significantly different from the sample population in regards to work function (Table 1), including a higher number of respondents with the most advanced paramedic training. Missing data amounted to 9% but were not predicted by any of the items within the survey, Little’s MCAR’s chi square (103) = 101.0, p = .538. This indicated that data were MCAR with no seeming bias detected in response patterns. Descriptive statistics (Table 1) showed that the sample consisted mainly of men with a majority of professional training of rescuer or above and a mean exposure to three to four critical events within the recent year. Level of PTS symptoms varied with a mean of 3.9 (4.7), with 30% of the sample reporting no symptoms at all.

**Table 1.** Descriptive statistics.

Factor		Sample	Population	Test statistic
Gender				χ^2^(1) = 1.5, p = .215
	Female	18.2% (n = 74)	17.5% (n = 129)	
	Male	80.3% (n = 327)	82.5% (n = 607)	
	Non-gender	0% (n = 0)		
	Other gender	0% (n = 0)		
	Missing	1.5% (n = 6)		
Work function				χ^2^(5) = 15.4, p = .009
	EMS transport personnel	6.9% (n = 28)	9.2% (n = 68)	
	Ambulance rescuer student	6.1% (n = 25)	8.5% (n = 63)	
	Ambulance rescuer assistant	13.8% (n = 56)	14.8% (n = 109)	
	Ambulance rescuer	36.4% (n = 148)	40.7% (n = 300)	
	Paramedic	34.4% (n = 140)	25.3% (n = 186)	
	Leader	2.5% (n = 10)	1.4% (n = 10)	
Area of service				χ^2^(7) = 11.7, p = .111
	Area 1	22.1% (n = 90)	22.4% (n = 165	
	Area 2	9.2% (n = 38)	11.1% (n = 82)	
	Area 3	16.3% (n = 66)	20.5% (n = 151)	
	Area 4	19.0% (n = 77)	14.3% (n = 105)	
	Area 5	9.7% (n = 40)	8.6% (n = 63)	
	Area 6	10.2% (n = 41)	12.2% (n = 90)	
	Area 7	9.0% (n = 37)	8.2% (n = 60)	
	Working across areas	4.5% (n = 18)	2.7% (n = 20)	
Age		40.4 (9.6)	–	–
Crisis support		4.5 (1.7)	–	–
Civil lifetime critical events		2.7 (2.4)	–	–
Work critical event recent year		3.7 (8.6)	–	–
Level of PTS		3.9 (4.7)		

EMS: emergency medical services; PTS: post-traumatic stress; SD: standard deviation.

### Categories of critical events

The raters identified a total of 32 categories. Only 6% of the items were categorised as non-events, where descriptions were mainly comments that did not describe events, for example: ‘*I meet a lot of taxing events but have been able to cope with these within my 20 years as a paramedic*’. Three categories did not describe operational events but rather frustrations regarding work, for example: ‘*general practitioners often call on an ambulance in a no emergency situation because they don’t have time to do home visits even though it’s part of their job*’. Overall, there was considerable variation in the number of respondents who endorsed the events in each category, ranging from 0.8% (n = 30) experiencing an operation with continued but meaningless resuscitation as critical, and 39% (n = 148) experiencing the death of a child as critical.

The focus group recognised and agreed on the extracted categories, indicating high face validity. They commented on the category of non-events and the three categories on frustration with the organisation, which they defined as non-critical events that should not be included in the scale. The final scale consisted of 28 categories (Table 2), and the interrater reliability was excellent, with Cohen’s kappa coefficient = 0.98, p < .001.

**Table 2.** The 28 categories of critical events identified by content analysis.

	Category of critical event	Percentage and number of respondents (n) reporting incidents
**1**	Suicide of children and youth	3.7% (n = 14)
**2**	Critical accident or illness in youth or children	31.4% (n = 119)
**3**	Death of children and youth	39% (n = 148)
**4**	Violence against children and youth	2.4% (n = 9)
**5**	Terminal illness in children and youth	5.8% (n = 22)
**6**	Severe neglect or social vulnerability in children and youth	5.8% (n = 22)
**7**	Suicide of adults	15% (n = 57)
**8**	Severe neglect and social vulnerability in adults	3.4% (n = 13)
**9**	Threats or violence against myself or colleague	28.0% (n = 106)
**10**	Risk of infections from serious diseases	1% (n = 4)
**11**	Own or colleague’s accident during service (e.g. driving accident)	2.1% (n = 8)
**12**	High-risk situations where you or your colleague were in danger	2.6% (n = 10)
**13**	Responding to a situation involving your relatives, friends or colleagues	2.9% (n = 11)
**14**	Abusive acts from colleague or patient during an operation	4.0% (n = 15)
**15**	Death or serious accidents which you identified with your own life	4.2% (n = 16)
**16**	Accidents with very severe or multiple injured or dead persons (e.g. train accidents, large traffic accidents, fires or work accidents)	28.8% (n = 109)
**17**	Simple or minor accidents with very severe consequences or deaths	12.4% (n = 47)
**18**	Adults with terminal illness / palliative transports	5% (n = 19)
**19**	Handling patient’s relatives in crisis or with strong emotional reactions	26.6% (n = 101)
**20**	Injuries or death caused by intentional acts of others (e.g. violence, homicide, rape or dangerous driving)	11.1% (n = 42)
**21**	Working with psychiatric patients who are vulnerable, erratic or aggressive	8.2% (n = 31)
**22**	Working with mutilated bodies (e.g. collecting body parts after accidents or homicide, burnt or decaying corpses, etc.)	3.4% (n = 13)
**23**	Working in a crowd monitoring you or interfering with your work	1.2% (n = 5)
**24**	Sudden or unexpected deaths (e.g. due to blood clot or apoplexia)	4.5% (n = 17)
**25**	Birth complications	0.8% (n = 3)
**26**	Repeated but meaningless resuscitation attempt	0.8% (n = 3)
**27**	Serious situation with lack of equipment or professional expertise (causing feelings of inadequacy and helplessness)	10% (n = 38)
**28**	Error made by you, your colleague or the responder system, causing damage to the patient	3.1% (n = 12)

### Predictive qualities of the Critical Incidents Scale for Ambulance Work – Denmark

Visual assessment of residual plots did not raise concern in regards to homoscedasticity nor in relation to linearity. The outcome of PTS was right skewed, which was accounted for by bootstrapping. There was no indication of multi-collinearity, with variance inflation factor ranging from 1.0 to 3.4. The full model significantly explained 17% of the variance of PTS. Also, inclusion of frequency of critical work incidents within the recent year significantly increased variance explained: adjusted R square = 0.17; R square change = 0.04 F_change_ (1, 303) = 13.5, p < .001. Work critical incidents within the recent year showed significant prediction of PTS symptoms (Table 3) with low to moderate effect size. Also, gender, age, crisis support and civil lifetime critical events significantly predicted PTS (Table 3).

**Table 3.** Predictive qualities of the Critical Incidents Scale for Ambulance Work – Denmark.

Factor	Coefficient beta	Standard error	Standardised coefficient beta	t-statistics	BCA level of significance
**Step 1**					
Constant	6.57	2.30		2.85	.005
Gender	1.49	0.60	0.14*	2.47	.014
Age	−0.09	0.04	−0.20*	−2.08	.038
Work function	0.05	0.24	0.01	0.23	.820
Seniority	0.09	0.05	0.20	1.95	.052
Service area	0.03	0.10	0.01	0.24	.812
Crisis support	−0.13	0.03	−0.22**	−4.01	< .001
Civil lifetime critical events	0.36	0.10	0.20**	3.73	< .001
**Step 2**					
Constant	5.91	2.26		2.61	.009
Gender	1.34	0.59	0.13*	2.25	.025
Age	−0.09	0.04	−0.20*	−2.13	.034
Work function	0.08	0.23	0.02	0.34	.733
Seniority	0.09	0.04	0.20*	1.98	.048
Service area	0.06	0.10	0.03	0.58	.561
Crisis support	−0.12	0.03	−0.20**	−3.78	< .001
Civil lifetime critical events	0.34	0.10	0.19**	3.57	< .001
Work critical event recent year	0.11	0.03	0.20**	3.67	< .001

*values significant at p < .05; **values significant at p < .001. BCA: bias corrected accelerated.

## Discussion

The current study showed that a scale of 28 categories could exhaustively describe 1092 qualitative descriptions of critical events in ambulance work.

The study showed that the frequency of critical events within the recent year significantly predicted PTS, even when adjusted for other well-known risk factors. The results indicate not only that the 28 categories are positively associated with PTS, but that repeated exposure to these categories of events constitutes a cumulative strain that is associated with increased levels of PTS.

The 28 categories of the Critical Incidents Scale for Ambulance Work – Denmark (CISAW-D) were comparable to categories identified in two similar studies on samples of US (Donnelly & Bennett, 2014) and German (Behnke et al., 2019) ambulance workers. A total of 21 of the 28 categories in the CISAW-D (items 2, 3, 4, 5, 6, 7, 8, 9, 10, 11, 12, 13, 15, 16, 17, 18, 20, 21, 22, 27, 28) were directly comparable to both or one of the scales from the German and the US samples. However, some of the categories in the CISAW-D were differentiated on subcategories in the US and German scales. For example, the CISAW-D category ‘*responding to a situation involving your close relatives, friends or colleagues*’ was described in two categories in the German study as ‘*an operation that included the interaction with a patient whose family you were personally acquainted with*’ and ‘*an operation that included an interaction with a patient who was a friend of yours*’. Similarly, the CISAW-D category ‘*injuries or deaths caused by intentional acts of others (e.g. violence, homicide, rape or insane driving, etc.)*’ contained two categories from the German scale: ‘*taking care of victims of severe violent crimes (e.g. murder, rape)*’ and ‘*taking care of victims of minor crimes (e.g. affray, stabbing)*’ and two from the US scale: ‘*encountered an adult who had been badly beaten*’ and ‘*encountered an adult who had been sexually assaulted*’.

Five categories from the CISAW-D had no direct overlap to the US and German categories but still had similarity to these. For example, the CISAW-D category ‘*handling patient relatives in crisis or with strong emotional reactions*’ was similar to the category in the German study ‘*an operation that included the bearing of bad news to a patient’s relatives*’ and the category in the US study ‘*made a death notification*’, since all entailed situations demanding the worker to contain strong emotional reactions from relatives. Only two categories from the present study had no similarity with categories from the other studies: ‘*continued but meaningless resuscitation attempts*’ and ‘*birth complications*’.

The similarity between categories in the German, US and Danish scales indicates that the CISAW-D represents critical events in ambulance work that are generally emotionally taxing. The study results also support the proposition that daily operational work that is not traumatic in the sense of being life-threatening or including the deaths of others might act as cumulative stressors that are associated with increased levels of PTS symptoms. The high similarity between the US, German and Danish samples also indicates that CISAW-D can be used as a screening tool in research or preventive initiatives focusing on exposure to critical events in ambulance work.

## Limitations

Sampling bias might be a limitation of the present study. Approximately 55% of the invited personnel responded to the questionnaire and although tests only showed systematic differences in regards to work function, possible unmeasured factors could have impacted the sample composition. For example, employees with existing mental health problems could have refrained from participating, which would likely lead to underreporting of some types of critical events. Considering the higher prevalence of paramedics in the sample compared to the sample population, the CISAW-D might over-represent categories of events deemed critical to this group of ambulance employees. It is possible that inclusion of more respondents with job functions based on lesser training, for example emergency medical services transport personnel or recruits, could have led to other findings. However, considering the degree of similarity between the Danish, German and US samples, it is unlikely that the CISAW-D categories are strongly affected by sampling bias.

The explorative design of the present study affected the choice of statistical analyses. It was only possible to assess frequency of overall number of events and not the frequency of each category of events separately. Indeed, the predictive validity of the categories may have been driven by the frequency of a specific group of the critical events in the CISAW-D. This should be considered in future studies using the CISAW-D, allowing for the respondents to address the frequency of each category separately. Similarly, future studies are needed to assess the predictive properties of the CISAW-D in a prospective design. The cross-sectional assessment of the association between frequency of exposure and PTS symptoms did not allow for considerations of the temporal dynamics between exposure and symptomatology.

## Practical implications

The CISAW-D can improve future initiatives of prevention of mental health problems in ambulance work. Screening for exposure frequency based on a formal and validated measure enables the construction of a preventive strategy involving contact and offers of psychosocial support to employees that are exposed and to initiate further support or rotation programmes for restitution. Early detection and prevention initiatives based on exposure screening are likely a more effective strategy compared to depending on employees to report their symptoms or ask for help, considering that symptom normalisation and fear of stigmatisation are known problems within this job type (McFarlane, 2014).

Systematic exposure-screening could be used as a supplement to other existing preventive measures such as debriefing or crisis intervention that are typically initiated when encountering adverse events outside the spectrum of daily operative situations. Relying on these acute interventions is probably insufficient since the current study as well as the studies by Donnelly and Bennett (2014) and Behnke et al. (2019) indicate that daily operative work events of ambulance personnel are experienced as critical incidents and are also a risk factor for the development of PTS symptoms.

## Conclusion

The present study developed a 28-item scale for critical events among Danish ambulance workers. The CISAW-D is a promising screening tool for exposure to critical events among ambulance personnel and a predictor of PTS symptomatology. The scale should be considered as a screening tool for critical events in future research on cumulative strain and post-traumatic symptoms among ambulance personnel.

## Acknowledgements

We thank Ambulance Syd for their co-operation on the data collection that enabled the current study.

## Author contributions

JP-T NBH, MLV and SRM contributed to the conceptualisation and design of the study. JP-T collected the data and NBH and JP-T analysed and interpreted the data. JP-T made the first article draft and JP-T, MLV, SRM and NBH made critical revisions to the manuscript. All authors approved the final manuscript, and all authors agree on full accountability of the content of the manuscript. JP-T acts as the guarantor for this article.

## Conflict of interest

None declared.

## Ethics

The study was presented to the Scientific Ethics Committee, receiving the formal response that according to Danish law, the study was not subject to approval by the committee (case # 20222000-78). Respondents participated based on informed and written consent. Procedures for treatment of data were approved by the Danish Data Protection Agency, journal # 20/47381.

## Funding

The study was funded by the Danish Work Environment Research Fund journal # 20-2022-03 20225100185 and the research fund of Region of Southern Denmark journal # 22/8605 efond1398.

## References

[bibr_1] Behnke A. Rojas R. Karrasch S. Hitzler M. & Kolassa I.-T. (2019). Deconstructing traumatic mission experiences: Identifying critical incidents and their relevance for the mental and physical health among emergency medical service personnel. *Frontiers in Psychology*, 10, 2305. https://doi.org/10.3389/fpsyg.2019.02305. 31695639 10.3389/fpsyg.2019.02305 PMC6817588

[bibr_2] Bonde J. P. E. Høy Jensen J. Smid G. E. Flachs E. M. Elklit A. Mors O. & Videbech P. (2022). Time course of symptoms in posttraumatic stress disorder with delayed expression: A systematic review. *Acta Psychiatrica Scandinavica*, 145(2), 116–131. 34523121 10.1111/acps.13372 PMC9293462

[bibr_3] Brewin C. R. (2003). *Posttraumatic stress disorder: Malady or myth*? Yale University Press.

[bibr_4] Brewin C. R. Andrews B. & Valentine J. D. (2000). Meta-analysis of risk factors for posttraumatic stress disorder in trauma-exposed adults. *Journal of Consulting and Clinical Psychology*, 68(5), 748–766. 11068961 10.1037//0022-006x.68.5.748

[bibr_5] Cloitre M. Shevlin M. Brewin C. R. Bisson J. I. Roberts N. P. Maercker A. Karatzias T. & Hyland P. (2018). The international trauma questionnaire: Development of a self-report measure of ICD-11 PTSD and complex PTSD. *Acta Psychiatrica Scandinavica*, 138(6), 536–546. 30178492 10.1111/acps.12956

[bibr_6] Donnelly E. A. & Bennett M. (2014). Development of a critical incident stress inventory for the emergency medical services. *Traumatology: An International Journal*, 20(1), 1–8.

[bibr_7] Elklit A. Pedersen S. S. & Jind L. (2001). The crisis support scale: Psychometric qualities and further validation. *Personality and Individual Differences*, 31(8), 1291–1302.

[bibr_8] Harvey S. B. Devilly G. J. Forbes D. Glozier N. McFarlane A. Phillips J. Sim M. Steel Z. & Bryant R. (2015). *Expert guidelines: Diagnosis and treatment of post-traumatic stress disorder in emergency service workers*. Report endorsed by the Royal Australian and New Zealand College of Psychiatrists. https://www.blackdoginstitute.org.au/wp-content/uploads/2020/04/ptsd_expert-guidelines_2015-1.pdf.

[bibr_9] Haugen P. T. Evces M. & Weiss D. S. (2012). Treating posttraumatic stress disorder in first responders: A systematic review. *Clinical Psychology Review*, 32(5), 370–380. 22561967 10.1016/j.cpr.2012.04.001

[bibr_10] Hsieh H. F. & Shannon S. E. (2005). Three approaches to qualitative content analysis. *Qualitative Health Research*, 15(9), 1277–1288. 16204405 10.1177/1049732305276687

[bibr_11] Kessler R. C. Sonnega A. Nelson C. B. & Bromet E. (1995). Posttraumatic stress disorder in the National Comorbidity Survey. *Archives of General Psychiatry*, 52(12), 1048–1060. 7492257 10.1001/archpsyc.1995.03950240066012

[bibr_12] King L. A. King D. W. Vogt D. S. Knight J. & Samper R. E. (2006). Deployment risk and resilience inventory: A collection of measures for studying deployment-related experiences of military personnel and veterans. *Military Psychology*, 18(2), 89–120.

[bibr_13] Liu H. Petukhova M. V. Sampson N. A. Aguilar-Gaxiola S. Alonso J. Andrade L. H. Bromet E. J. de Girolamo G. Haro J. M. Hinkov H. Kawakami N. Koenen K. C. Kovess-Masfety V. Lee S. Medina-Mora M. E. Navarro-Mateu F. Neill S. Piazza M. Posada-Villa J. … World Health Organization World Mental Health Survey Collaborators. (2017). Association of DSM-IV posttraumatic stress disorder with traumatic experience type and history in the World Health Organization World Mental Health Surveys. *JAMA Psychiatry*, 74(3), 270–281. 28055082 10.1001/jamapsychiatry.2016.3783 PMC5441566

[bibr_14] McFarlane A. C. (2012). The occupational implication of the prolonged effects of repeated exposure to traumatic stress. In R. Hughes A. Kinder & L. C. Cooper (Eds.), *International handbook of workplace trauma support* (1st ed., pp. 121–138). John Wiley & Sons, Ltd.

[bibr_15] McFarlane A. C. (2014). PTSD and DSM-5: Unintended consequences of change. *The Lancet Psychiatry*, 1(4), 246–247. 26360842 10.1016/S2215-0366(14)70321-9

[bibr_16] Mitchell J. T. (1983). When disaster strikes … the critical incident stress debriefing process. *Journal of Emergency Medical Services*, 8(1), 36–39. 10258348

[bibr_17] Monnier J. Cameron R. P. Hobfoll S. E. & Gribble J. R. (2002). The impact of resource loss and critical incidents on psychological functioning in fire-emergency workers: A pilot study. *International Journal of Stress Management*, 9(1), 11–29.

[bibr_18] Petrie K. Milligan-Saville J. Gayed A. Deady M. Phelps A. Dell L. Forbes D. Bryant R. A. Calvo R. A. Glozier N. & Harvey S. B. (2018). Prevalence of PTSD and common mental disorders amongst ambulance personnel: A systematic review and meta-analysis. *Social Psychiatry and Psychiatric Epidemiology*, 53(9), 897–909. 29869691 10.1007/s00127-018-1539-5

[bibr_19] Skogstad M. Skorstad M. Lie A. Conradi H. S. Heir T. & Weisæth L. (2013). Work-related post-traumatic stress disorder. *Occupational Medicine*, 63(3), 175–182. 23564090 10.1093/occmed/kqt003

[bibr_20] Utzon-Frank N. Breinegaard N. Bertelsen M. Borritz M. Eller N. H. Nordentoft M. Olesen K. Rod N. H. Rugulies R. & Bonde J. P. (2014). Occurrence of delayed-onset post-traumatic stress disorder: A systematic review and meta-analysis of prospective studies. *Scandinavian Journal of Work, Environment and Health*, 40(3), 215–229. 10.5271/sjweh.3420 24599261

[bibr_21] Weiss D. S. Brunet A. Best S. R. Metzler T. J. Liberman A. Pole N. Fagan J. A. & Marmar C. R. (2010). Frequency and severity approaches to indexing exposure to trauma: The critical incident history questionnaire for police officers. *Journal of Traumatic Stress*, 23(6), 734–743. 21171134 10.1002/jts.20576 PMC3974917

